# Insights into the identification of a molecular signature for amyotrophic lateral sclerosis exploiting integrated microRNA profiling of iPSC-derived motor neurons and exosomes

**DOI:** 10.1007/s00018-022-04217-1

**Published:** 2022-03-14

**Authors:** Mafalda Rizzuti, Valentina Melzi, Delia Gagliardi, Davide Resnati, Megi Meneri, Laura Dioni, Pegah Masrori, Nicole Hersmus, Koen Poesen, Martina Locatelli, Fabio Biella, Rosamaria Silipigni, Valentina Bollati, Nereo Bresolin, Giacomo Pietro Comi, Philip Van Damme, Monica Nizzardo, Stefania Corti

**Affiliations:** 1 Foundation IRCCS Ca’ Granda Ospedale Maggiore Policlinico, Neurology Unit, Milan, Italy; 2 Department of Physiopathology and Transplants, Dino Ferrari Center, University of Milan, via Francesco Sforza 35, 20122 Milan, Italy; 3 EPIGET LAB, Unit of Occupational Medicine, Department of Clinical Sciences and Community Health, University of Milan, IRCCS Ca’ Granda Foundation Ospedale Maggiore Policlinico, Milan, Italy; 4 Department of Neurosciences, Laboratory of Neurobiology, Center for Brain and Disease, KU Leuven, University of Leuven, Leuven, Belgium; 5 Neurology Department, University Hospitals Leuven, Leuven, Belgium; 6 Department of Neurosciences, Laboratory for Molecular Neurobiomarker Research, Leuven Brain Institute, KU Leuven, 3000 Leuven, Belgium; 7 Laboratory of Medical Genetics, IRCCS Ca’ Granda Foundation Ospedale Maggiore Policlinico, Milan, Italy; 8 Foundation IRCCS Ca’ Granda Ospedale Maggiore Policlinico, Neuromuscular and Rare Diseases Unit, Milan, Italy

**Keywords:** ALS, miRNA, Motor neurons, Exosomes, CSF

## Abstract

Amyotrophic lateral sclerosis (ALS) is a rare neurodegenerative disorder characterized by progressive degeneration of motor neurons (MNs). Most cases are sporadic, whereas 10% are familial. The pathological mechanisms underlying the disease are partially understood, but it is increasingly being recognized that alterations in RNA metabolism and deregulation of microRNA (miRNA) expression occur in ALS. In this study, we performed miRNA expression profile analysis of iPSC-derived MNs and related exosomes from familial patients and healthy subjects. We identified dysregulation of miR-34a, miR-335 and miR-625-3p expression in both MNs and exosomes. These miRNAs regulate genes and pathways which correlate with disease pathogenesis, suggesting that studying miRNAs deregulation can contribute to deeply investigate the molecular mechanisms underlying the disease. We also assayed the expression profile of these miRNAs in the cerebrospinal fluid (CSF) of familial (fALS) and sporadic patients (sALS) and we identified a significant dysregulation of miR-34a-3p and miR-625-3p levels in ALS compared to controls. Taken together, all these findings suggest that miRNA analysis simultaneously performed in different human biological samples could represent a promising molecular tool to understand the etiopathogenesis of ALS and to develop new potential miRNA-based strategies in this new propitious therapeutic era.

**Supplementary Information:**

The online version contains supplementary material available at 10.1007/s00018-022-04217-1.

## Introduction

Amyotrophic lateral sclerosis (ALS) is a complex multifactorial neurodegenerative disease characterized by progressive degeneration of upper and lower motor neurons (MNs) in the brain and spinal cord, leading to progressive muscle paralysis and precocious death [1]. Except for riluzole and, more recently, edaravone, which only modestly increase survival, there is no specific treatment for ALS [2]. Most cases are sporadic (sALS), while only 10% of ALS patients have a family history of disease and exhibit familial ALS (fALS) [1]. The most common genetic causes of ALS are the hexanucleotide repeat expansion (HRE) in *Chromosome 9 open reading frame 7*2 (*C9orf72*) gene [3, 4] and mutations in *superoxide dismutase 1* (*SOD1*) [5], *TAR DNA binding protein 43* (*TARDBP*) [6] or *Fused in sarcoma* (*FUS*) [7, 8]. Although several genes have been linked to alterations of protein quality control systems, perturbation of cytoskeletal dynamics in axons and RNA metabolism [9], the pathological mechanisms underlying the disease remain partially understood. Interestingly, both *TARDBP* and *FUS* are involved in RNA processing, including microRNA (miRNA) biogenesis, sequestration or repression [10–18]. MiRNAs are small RNA molecules (~ 20 nucleotides) that play a key role as endogenous regulators of gene expression. Indeed, they can act at the post-transcriptional level by promoting degradation or translational repression of target messenger RNAs (mRNAs) [19]. Since each miRNA can regulate hundreds of target mRNAs, alterations in the miRNA expression profile can modulate entire gene networks, potentially modifying the pathogenesis of complex syndromes such as neurodegenerative disorders. Interestingly, since approximately 70% of miRNAs are expressed in the brain [20, 21], they are probably involved in the majority of the pathogenetic mechanisms of neurodegeneration [22]. In addition, a small population of miRNAs was detected to circulate in exosomes, small extracellular vesicles (EVs) that can act as mediators of cell-to-cell communication by transferring their cargos to both neighboring and distant cells [23–26].

It has already been described that a dysregulation of miRNA expression occurs in ALS [27–29], but the downstream pathological events associated with MN degeneration have not been completely clarified yet. Here, we aim to shed light on the common molecular pathways associated with MN degeneration among forms of ALS with different genetic backgrounds. We assessed the miRNA expression profiles of *C9orf72-*, *SOD1-* and *TARDBP*- iPSC-derived MNs, as well as the expression levels of exosomal miRNAs (ex-miRNAs) and we identified a small subset of miRNAs dysregulated in both MNs and exosomes. We further investigated the expression level of these miRNAs in the cerebrospinal fluid (CSF) of ALS patients to identify a miRNA signature shared across different forms of the disease. Noteworthy, altered expression of these molecules in the CSF may represent a useful hallmark of neurodegenerative diseases since miRNAs can closely mirror the physiological and pathological conditions of the central nervous system (CNS) [30].

This is the first work which analyzed simultaneously miRNAs isolated from different human biological samples, such as MNs, exosomes and CSF of different ALS types, offering an innovative approach to investigate the molecular bases of the disease. These findings could provide significant insights into ALS pathogenesis, contributing to translation into the clinic of new potential miRNA-based therapeutic strategies [31–34].

## Materials and methods

### iPSC generation and differentiation into MNs

Fibroblasts derived from skin biopsies of ALS patients (*n* = 2 *SOD1*, p.A4V and p.L144F; *n* = 2 *TARDBP*, p.G287S and p.G294V; *n* = 2 *C9orf72*, 583 repeats and 917 repeats) and healthy subjects (*n* = 3) were reprogrammed into induced Pluripotent Stem Cells (iPSCs) using the CytoTune®-iPS 2.0 Sendai Reprogramming Kit (ThermoFisher Scientific). Karyotype analyses were performed to evaluate genetic stability. iPSCs were stained for specific stem cell markers and differentiated into MNs following the protocol described by Maury and colleagues [35]. The proper phenotype was assessed with immunostaining for typical MN markers.

### qPCR analysis

Total RNA was extracted from each MN sample using ReliaPrep™ RNA Cell Miniprep System kit (Promega) and reverse transcribed using the TaqMan® MicroRNA Reverse Transcription Kit (ThermoFisher Scientific) and First Strand cDNA Synthesis kit (GE Healthcare). Exosomes were collected by ultracentrifugation and assessed by the NanoSight NS300 System (Malvern Panalytical). ex-miRNA extraction was performed with the combination of miRNeasy kit and RNeasy Cleanup Kit (Qiagen) and assessed through the 2100 Bioanalyzer RNA system (Agilent Technologies). Reverse transcription of ex-miRNA was followed by preamplification with TaqMan® Preamp Master Mix kit (ThermoFisher Scientific). Gene and miRNA expression levels were assayed on the 7500 Real Time PCR System (Applied Biosystem). Relative expression quantification was performed by the 2^(-ΔΔCt) method, using 18S or RNU6 as reference. All data are mean of triplicates. Only genes and miRNAs with Ct < 35 were taken into consideration for subsequent analysis. All the TaqMan® assay IDs are available upon request.

### Microfluidic cards assay

TaqMan® Low Density Arrays (TLDA, ThermoFisher Scientific) were used for miRNA profiling. Plates were run on 7900HT Fast Real Time PCR System (Applied Biosystem) and quantification was performed with the Gene Expression Suite Software (ThermoFisher Scientific), using RNU6 as control, automatic baseline settings and a threshold of 0.2. For ex-miRNAs, data were normalized using the global mean method [36].

### Bioinformatic analysis

miRTarBase and miRWalk were exploited to identify validated and predicted miRNA target genes and related pathways. Genes identified in both databases were processed with STRING (version 11) to predict functional interactions among targets, setting the threshold at 0.7 for interaction score and excluding interaction predicted by gene fusion. Cytoscape (version 3.7.2) was used to visualize complex miRNA-gene networks as well as functional interactions. GTex database (Release 8 since Aug 26, 2019) was employed to assay the tissue-specificity gene expression levels. Molecular pathway analysis to identify enriched pathways was performed against Reactome (version 72 on Apr 27, 2020) correcting p-values for multiple testing (Benjamini–Hochberg).

### MN treatment with miRNA mimics

ALS-MNs were transfected with a combination of mirVana® miRNA mimics hsa-miR-335-3p (MC13018), hsa-miR-335-5p (MC10063), hsa-miR-34a-3p (MC13089) and hsa-miR-34a-5p (MC10063) by using Lipofectamine® RNAiMAX Transfection Kit (ThermoFisher Scientific) to a final total concentration of 15 nM for 48 h.

### Analysis of miRNA isolated from CSF

Circulating miRNAs were isolated from 300 μL of cerebrospinal fluid (CSF) using NucleoSpin® miRNA plasma kit (Macherey Nagel). RT reactions were performed using 10 ng of RNA, preamplified and run on the 7900HT Fast Real Time PCR System (Applied Biosystem). The expression level of each miRNA was normalized to the average levels of hsa-miR-125b [37] and referred to control samples. Relative expression quantification was performed by the 2^(−ΔCt) method, where ΔCt = Ct miR-X − Ct miR-125b. All data are mean of triplicates. Only miRNAs with Ct < 35 were taken into consideration for the analysis.

### Patient evaluation

ALS patients and healthy controls (HC) were recruited at the Neurology Unit of Fondazione IRCCS Ca’ Granda Ospedale Maggiore Policlinico of Milan and at the Neurology Department of the University Hospitals in Leuven. ALS diagnosis was formulated according to the El Escorial Revised and Awaji-Shima diagnostic criteria and lumbar puncture for CSF collection was performed during the diagnostic assessment. Site and symptom onset were defined according to the first patient-reported weakness, and disease duration was calculated at the time of CSF collection. HC were defined as individuals with possible neurological symptoms, without evidence of underlying neurological disease after an appropriate diagnostic assessment.

### Statistical analysis

Two-tailed, unpaired Student's *t* test was employed to compare the mean expression level of miRNAs detected in MNs and exosomes. q-PCR quantification was expressed as mean with SEM. Data were analyzed with GraphPad Prism Version 5. Baseline demographic and clinical features of ALS patients and HC were analyzed through descriptive statistics. After assessing for normality, continuous variables were reported as mean ± standard deviation (SD) or median and interquartile range [IQR]. Mann–Whitney and Kruskal–Wallis tests were employed to perform between-group comparisons and to compare CSF miRNA levels between ALS and healthy controls, and among disease groups. Receiver Operating Characteristic (ROC) curves were generated and area under the curve (AUC) was calculated to assess accuracy of CSF miRNA levels in discriminating between ALS and controls of between two ALS subgroups. The ratios of levels of CSF miRNAs were calculated for significant pairs [38]. Best cut-off values were calculated with Youden’s Index. Univariable binomial logistic regression models were employed to evaluate the association among CSF miRNA levels and the disease. Statistical analysis was performed with GraphPad Prism Version 9.1.

### Ethical statement

The studies involving human samples were conducted in accordance with the ethical standards of the Declaration of Helsinki and with national legislation and institutional guidelines. Human fibroblast cell lines were obtained from Eurobiobank with informed consent approved by the ethical committee at Fondazione IRCCS Ca' Granda Ospedale Maggiore Policlinico, Milan. All subjects provided written informed consent approved by the local ethical committee for the collection, storage and analysis of CSF samples (0004520, S50354, S55312, S59552). This experimental study was conducted in accordance with the international GLP and GCP guidelines.

## Results

### Dysregulation of miRNA expression in ALS-MNs affects molecular pathways associated with MN degeneration

#### miRNA profiling

**Fig. 1:**
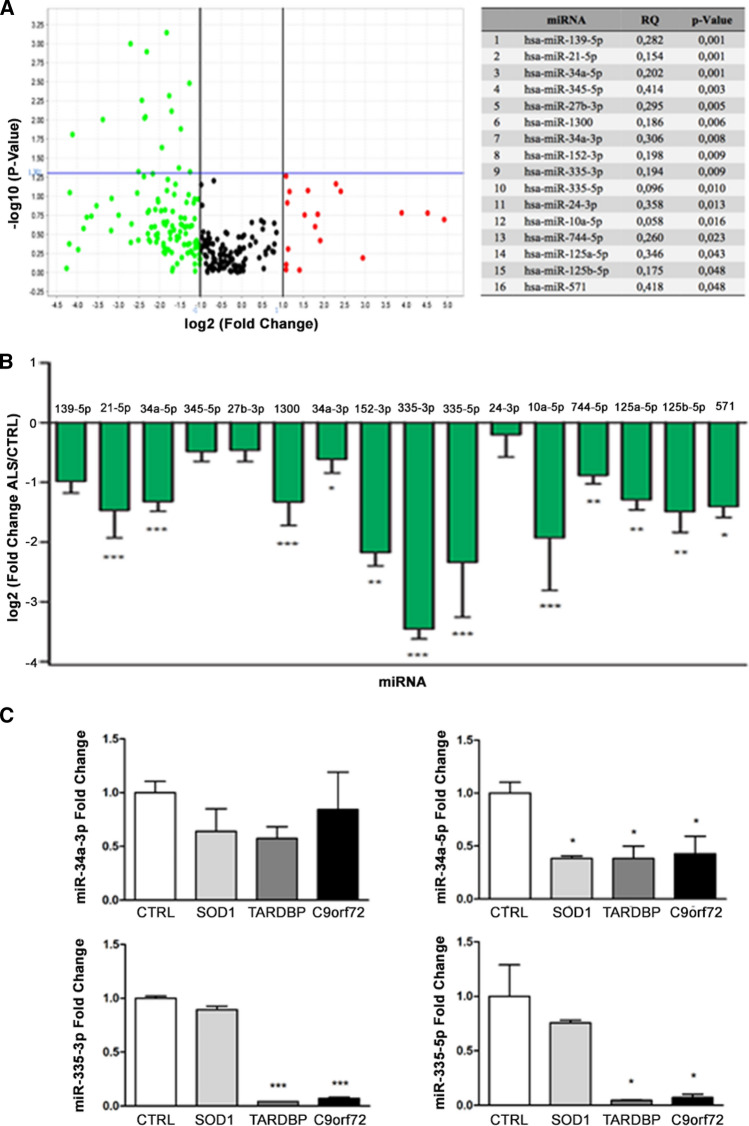
miRNA expression profiling of iPSC-derived MNs. **A** Volcano plot and list of dysregulated miRNAs. Results are presented enclosing ALS subjects presenting *C9orf72*, *TARDBP* or *SOD1* mutations in a unique biological group (*n* = 6) versus controls (*n* = 3). The statistically significant downregulated miRNAs are listed in the table. **B** Specific qPCR assays confirmed the downregulation of identified miRNAs in ALS-MNs (*n* = 6) compared to controls (*n* = 3) (*P < 0.05, **P < 0.01, ***P < 0.001, student *t*-test, values represent means + SEM). **C** qPCR assays confirmed the specific reduction of miR-34a-3p, miR-34a-5p, miR-335-3p or miR-335-5p expression in *C9orf72*-MNs, *SOD1*-MNs and *TARDBP*-MNs (at least *n* = 3 healthy subjects, *C9orf72*-MNs, *SOD1*-MNs and *TARDBP*-MNs, *P < 0.05, *** *P* < 0.001, student t-test, values represent means + SEM)

After generation of iPSCs (Fig. S1, A) and MNs (Fig. S1, B) of healthy individuals and ALS patients carrying *C9orf72*, *SOD1* and *TARDBP* mutations, we profiled the miRNA transcriptome of ALS-MNs by TaqMan® Low Density Arrays (TLDA). We identified a subset of 16 significantly downregulated miRNAs (Fig. 1A) whose reduced expression was validated by qPCR (Fig. 1B). Interestingly, when *C9orf72*-MNs, *SOD1*-MNs or *TARDBP*-MNs were considered independent biological groups, the profiling data showed that the expression of both miR-34a and miR-335 was reduced in all three MN lines (Table S1, A). This downregulation was validated by RT-PCR for miR-34a-3p, miR-34a-5p (*SOD1*-MNs: *P* < 0.05; *TARDBP*-MNs: *P* < 0.05; *C9orf72*-MNs: *P* < 0.05), miR-335-3p (*TARDBP*-MNs: *P* < 0.001; *C9orf72*-MNs: *P* < 0.001) and miR-335-5p (*TARDBP*-MNs: *P* < 0.05; *C9orf72*-MNs: *P* < 0.05) (Fig. 1C).

#### Bioinformatics

**Fig. 2:**
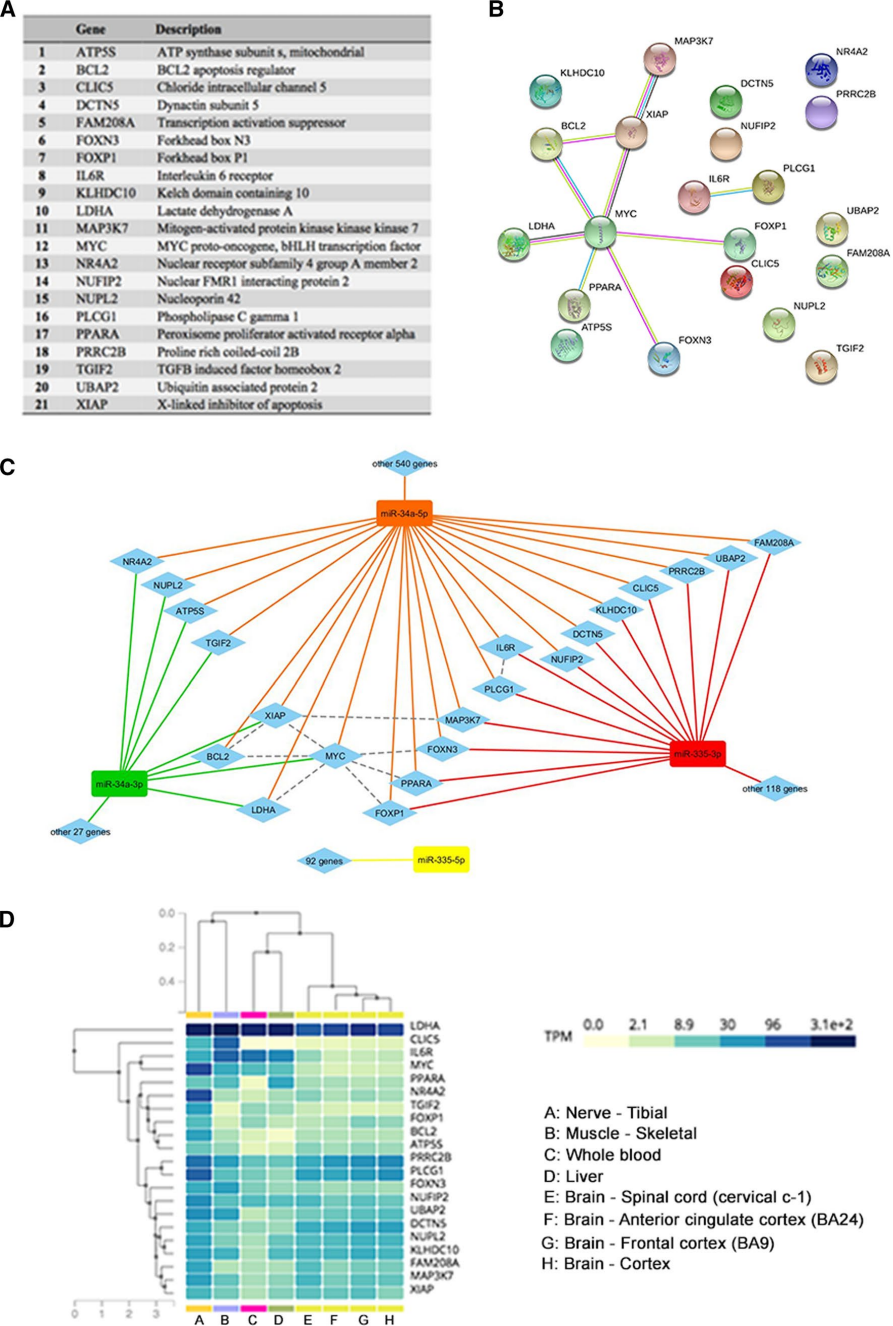
Bioinformatic analysis of dysregulated miRNAs in *C9orf72-*, *SOD1-* and *TARDBP*-MNs. **A** List of candidate genes identified by bioinformatic analysis. **B** STRING analysis disclosed the predicted functional interactions among target genes. **C** Cytoscape map of the miRNA-target genes interactions and functional connections among target genes. **D** GTEx plot displaying the tissue-specific expression of identified target genes, with a special focus on neural tissues

To identify target genes and related pathways associated with miR-34a-3p, miR-34a-5p, miR-335-3p and miR-335-5p, we employed the miRWalk and miRTarBase databases, which provide a list of validated and predicted miRNAs. We identified 21 genes targeted by at least two of these four miRNAs in both the databases, to obtain the most stringent and reliable dataset (Fig. 2A). Notably, these 21 genes were all targets of miR-34a-5p, while none of them was targeted by miR-335-5p. In particular, 13 out of these 21 genes were targeted by both miR-34a-5p and miR-335-3p, whereas the remaining 8 genes were targeted by both miR-34a-5p and miR-34a-3p. To predict the functional interactions among these target genes, the Search Tool for the Retrieval of Interacting Genes/Proteins (STRING) (Fig. 2B) and miRNA–gene interactions were used, as well as functional connections from STRING were visualized exploiting Cytoscape (Fig. 2C). We queried the Genotype-Tissue Expression (GTEx) database to assay the tissue-specific expression of these 21 genes, focusing on their expression in brain and spinal cord (Fig. 2D). Finally, we employed the Reactome Pathway Database to identify the biological pathways in which these genes are involved. Three hundred forty-three pathways (141 with statistical significance) were hit by at least one of 16 out of 21 genes (Table S2). In particular, the most enriched pathways are mainly involved in neurodegeneration processes, such as intrinsic pathway for apoptosis (*P* < 0.001), programmed cell death (*P* < 0.01), death receptor signaling (*P* = 0.05), cytokine signaling in the immune system (*P* < 0.001) and SUMOylation (*P* < 0.01). Due to the relevance of apoptosis and apoptotic pathways in MN degeneration, we selected *BCL2*, *IL6R*, *MAP3K7*, *PLCG1*, *PPARA* and *PRRC2B* as promising candidates for further investigations. We also investigated *LDHA* due to its very high expression levels in neural tissues. Finally, we investigated *FOXN3* given its crucial role in DNA damage [39] as well as *NUFIP2* for its interaction with FMRP protein, involved in synaptic plasticity [40].

#### miRNA mimic transfection

**Fig. 3:**
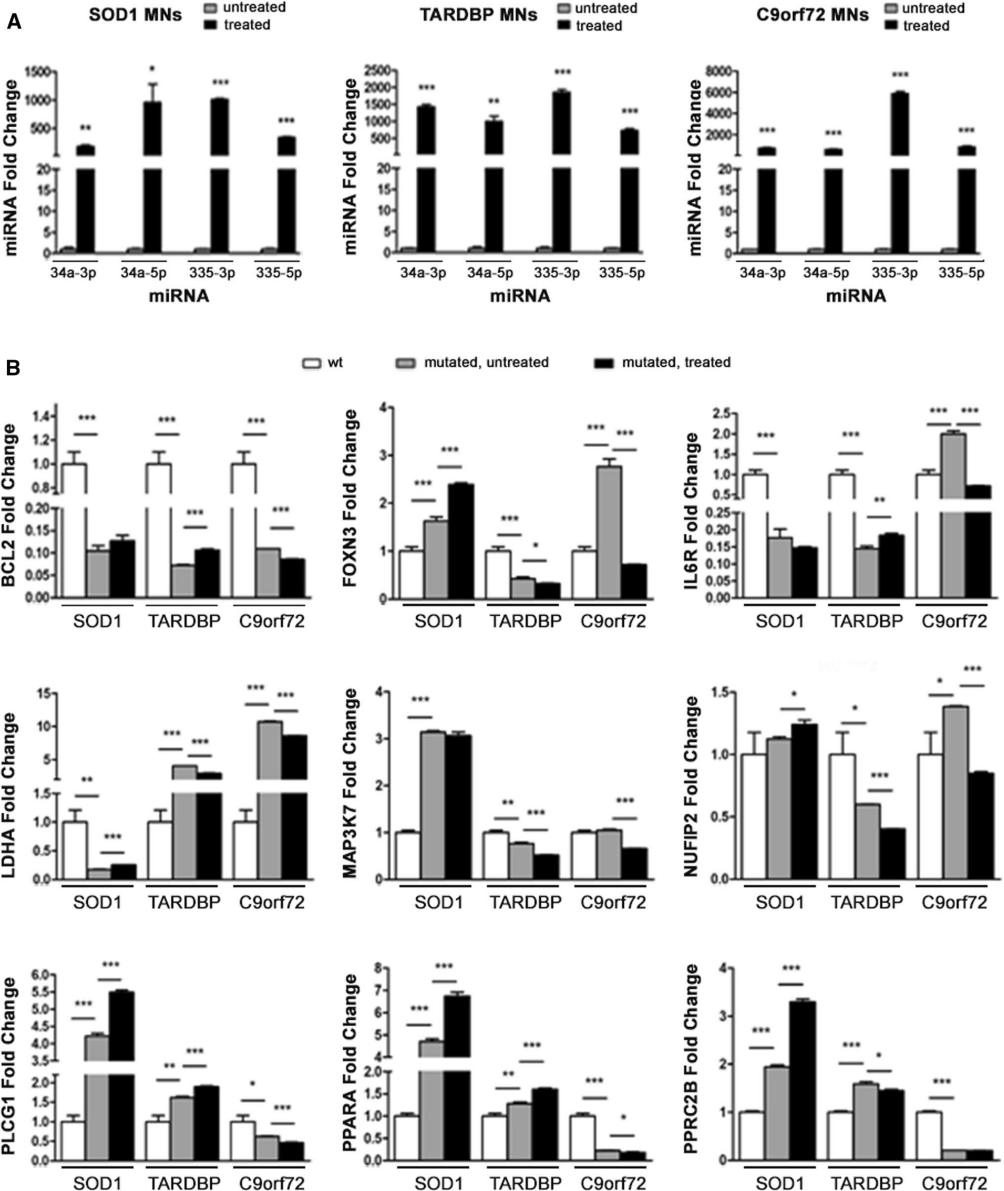
ALS-MNs transfection with miRNA mimics*.* **A** qPCR experiments showed the efficiency of transfection with the four selected miRNA mimics in *SOD1*-, *TARDBP*- and *C9orf72*-MNs (at least *n* = 3 healthy subjects, *C9orf72*-MNs, *SOD1*-MNs and *TARDBP*-MNs, *P < 0.05, **P < 0.01, *** *P* < 0.001, student *t*-test, values represent means + SEM). **B** qPCR experiments showed the dysregulation of all selected target genes in *SOD1*-, *TARDBP*- and *C9orf72*-MNs, which could not be completely rescue by the treatment with miRNA-mimics (at least *n* = 3 healthy subjects, *C9orf72*-MNs, *SOD1*-MNs and *TARDBP*-MNs, *P < 0.05, **P < 0.01, ***P < 0.001, student *t* test, values represent means + SEM)

We assayed whether synthetic sequences that mimic endogenous miRNAs can functionally rescue miRNA levels and modulate the expression of target genes of interest (GOI). To this end, we transfected ALS-MNs with the four miRNA mimics (Fig. 3A) and assessed the expression of GOI. Overall, our results showed a general dysregulation of almost all the selected target genes in affected MNs compared to controls, with notable differences among the expression levels of these genes in *C9orf72*-, *SOD1*- and *TARDBP*-MNs. However, the administration of miRNA mimics was not able to significantly modulate or completely rescue the expression of GOI (Fig. 3B).

### Exosomal miRNA profiling reveals their potential role in intercellular communication

#### ex-miRNAs profiling

**Fig. 4:**
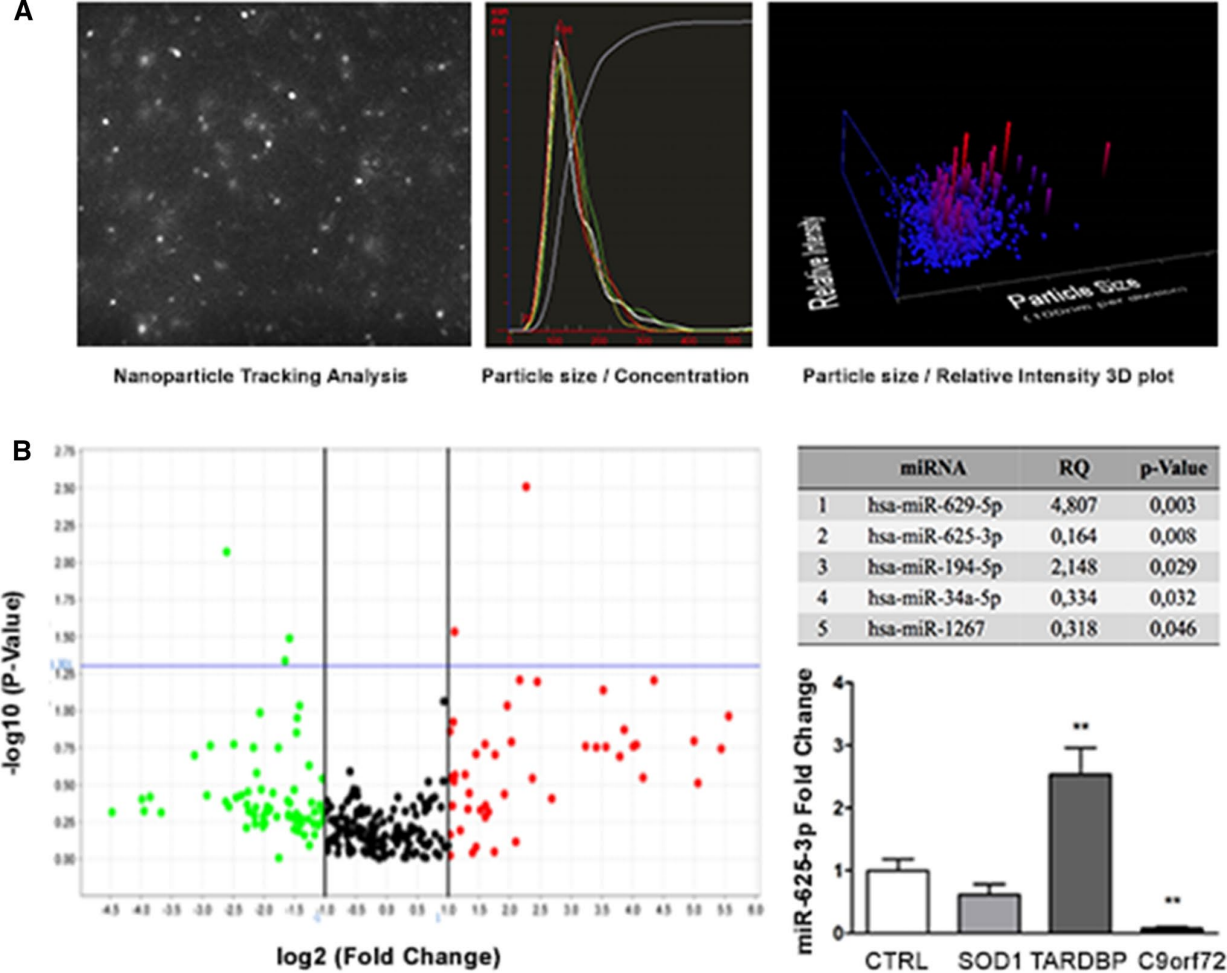
Exosomal miRNA profiling. **A** Representative plots of nanoparticle tracking analysis. **B** Volcano plot and list of dysregulated miRNAs identified in exosomes released from ALS-MNs. Results are presented enclosing ALS subjects in a unique biological group (*n* = 6) versus controls (*n* = 3). qPCR assays confirmed a statistically significant downregulation of miR-625-3p in exosomes isolated from *C9orf72*-MNs (at least *n* = 3, **P < 0.01), while there is a strong increase of its expression in exosomes derived from *TARDBP*-MNs (at least *n* = 3, **P < 0.01, student *t *test, values represent means + SEM)

The exosomal nature of EVs isolated from ALS-MN culture media was confirmed by nanoparticle tracking analysis (Fig. 4A). We profiled the expression levels of ex-miRNAs by TLDA. In particular, we found that miR-34a-5p, miR-625-3p and miR-1267 expression was downregulated while miR-629-5p and miR-194-5p levels were upregulated (Fig. 4B). Notably, miR-625-3p expression was always significantly downregulated in *C9orf72*- and *SOD1*-exosomes when considered as independent biological groups (Table S1, B). However, we confirmed by qPCR the dysregulation of ex-miR-625-3p expression only in exosomes isolated from *C9orf72* lines (*P* < 0.01) while *TARDBP* exosomes showed a strong increase in ex-miR-625-3p content (*P* < 0.01) (Fig. 4B).

#### Bioinformatics

**Fig. 5:**
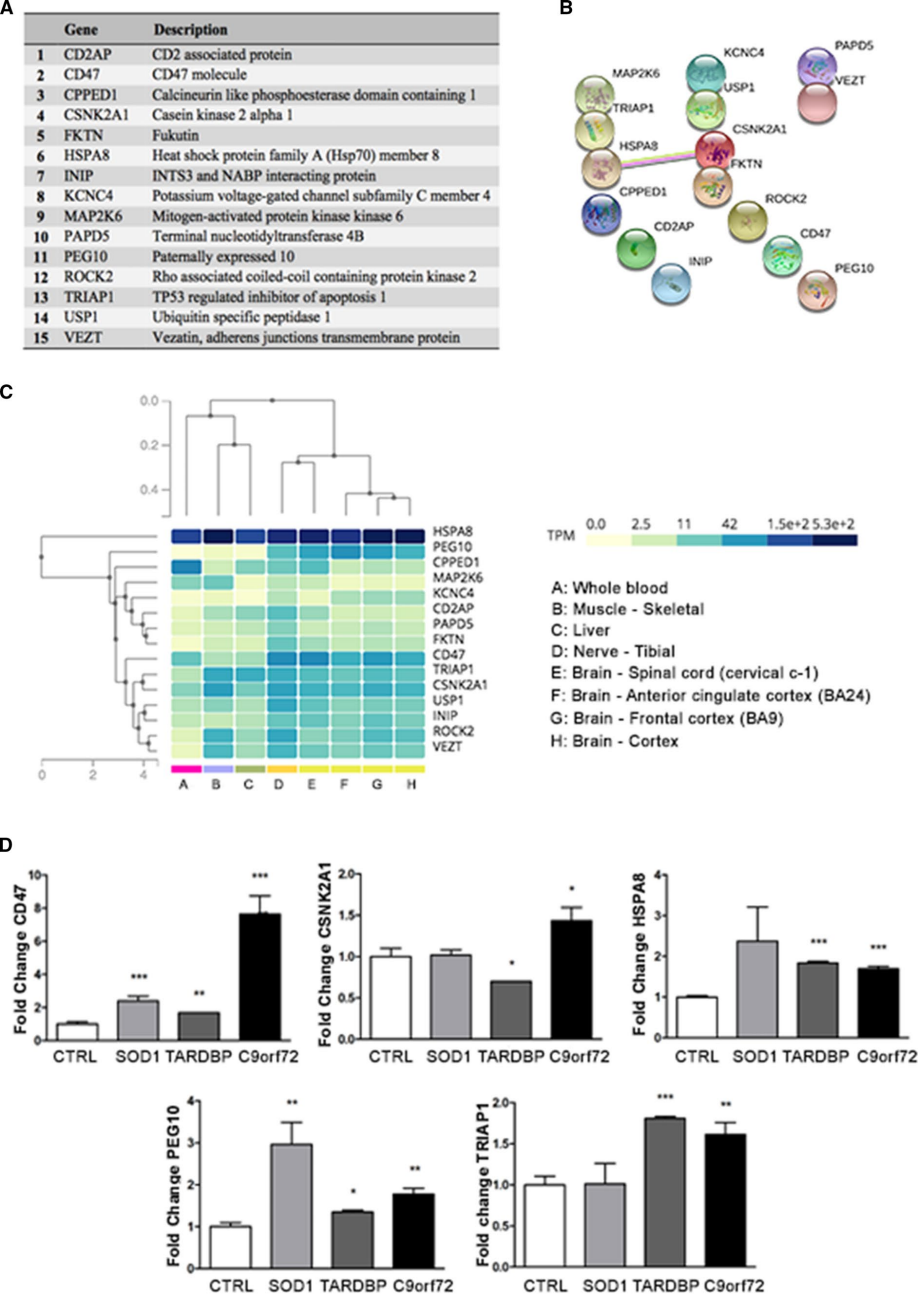
Bioinformatic analysis of ex-miR-625-3p. **A** List of predicted target genes of miR-625-3p. **B** STRING analysis showed the functional networks among the target genes. **C** GTEx chart illustrates the neural-specific expression of identified target genes. **D** qPCR assays revealed the dysregulation of *CD47*, *CSNK2A1*, *HSPA8*, *PEG10* and *TRIAP1* expression in almost all ALS-MNs (at least *n* = 3 healthy subjects, *C9orf72*-MNs, *SOD1*-MNs and *TARDBP*-MNs, *P < 0.05, **P < 0.01, ***P < 0.001, student *t *test, values represent means + SEM)

To deeply elucidate the role of ex-miR-625-3p as a potential mediator of intercellular communication, we performed bioinformatics analysis as previously described for MNs. We identified 15 genes as validated targets of miR-625-3p (Fig. 5A) and processed them with STRING to identify predicted functional interactions (Fig. 5B). We assessed their expression level with the GTEx database (Fig. 5C). Enrichment analysis revealed that 10 out of 15 identified genes were found in the Reactome Pathway Database, with 148 pathways being enriched for at least one of these genes (37 statistically significant, Table S3). Transcriptional regulation by TP53 as well as the role of TP53 in regulating the transcription of cell death genes, together with the cell–cell communication pathway, autophagy and axon guidance stood out significantly. Considering the biological relevance of these pathways together with the expression of selected genes in the CNS and putative interactions among them, we selected *CD47*, *CSNK2A1*, *HSPA8* and *TRIAP1* as promising candidates for further investigations. We include *PEG10* in the group of candidate genes since it has already been investigated as possibly involved in pathological mechanisms underlying ALS [41]. The dysregulation of predicted target genes in almost all ALS-MN lines suggests the potential role of miR-625-3p as a mediator of cell-to-cell communication (Fig. 5D).

### A subset of specific miRNAs is dysregulated in ALS-MNs and exosomes

**Fig. 6:**
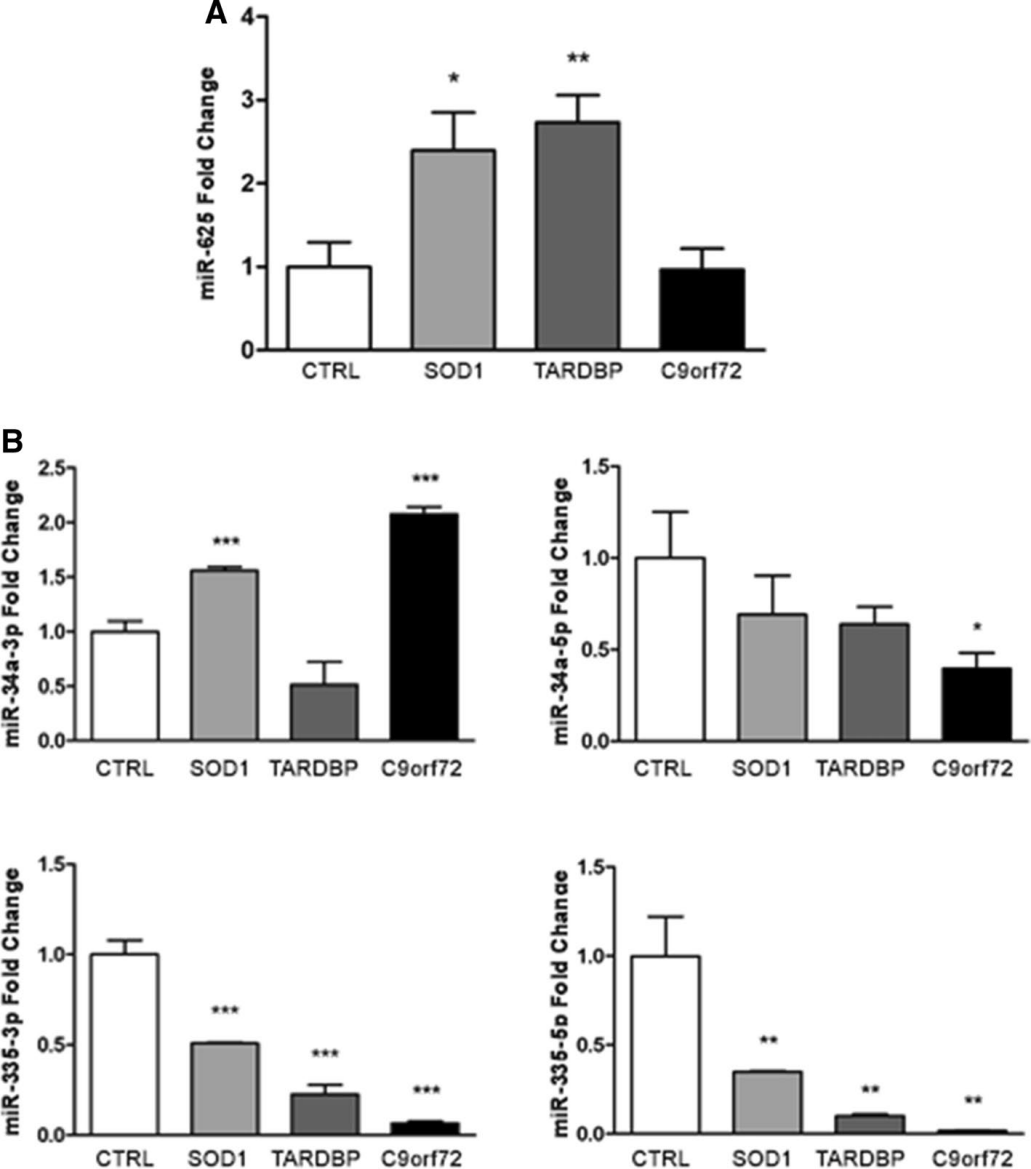
Correlation between profiling data of ALS-MNs and released exosomes. **A** qPCR assays confirmed the statistically significant dysregulation of miR-625-3p in *SOD1*-MNs and *TARDBP*-MNs (at least *n* = 3, *P < 0.05, **P < 0.01, student *t *test, values represent means + SEM). **B** qPCR experiments validated the deregulation of ex-miR-34a-3p, ex-miR-34a-5p, ex-miR-335-3p and ex-miR-335-5p expression in all exosomes isolated from affected MN lines (at least *n* = 3 healthy subjects, *C9orf72*-MNs, *SOD1*-MNs and *TARDBP*-MNs, *P < 0.05, **P < 0.01, ***P < 0.001, student *t *test, values represent means + SEM)

To correlate the miRNA profile data of MNs and related exosomes, we assessed the expression levels of miR-625-3p in ALS-MNs. Data from TLDA experiments showed a reduced expression of miR-625-3p in *C9orf72*- and *SOD1*-MNs, whereas no change was detected in *TARDBP*-MNs (Table S1, A). However, qPCR validation revealed significantly increased expression of this miRNA in *SOD1-*MNs (*P* < 0.05) and *TARDBP-*MNs (*P* < 0.001) (Fig. 6A).

Conversely, we assessed the expression levels of miR-34a-3p, miR-34a-5p, miR-335-3p and miR-335-5p in ALS-exosomes detecting an overall downregulation of all four miRNAs in exosomes released from *C9orf72*-, *SOD1*- and *TARDBP*-MNs compared to control exosomes (Table S1, B). qPCR validation assays revealed a general downregulation of ex-miR-34a-5p (*C9orf72*: *P* < 0.05), ex-miR-335-3p (*SOD1*, *TARDBP*, and *C9orf72*: *P* < 0.001) and ex-miR-335-5p (*SOD1*, *TARDBP*, and *C9orf72*: *P* < 0.01), along with an upregulation of ex-miR-34a-3p in *SOD1-* and *C9orf72*-MNs (*P* < 0.001) (Fig. 6B).

### A subset of miRNAs is increased in the CSF of some ALS forms

We investigated the expression of the previously identified miRNAs in the CSF of a cohort of ALS patients and HC. We enrolled 55 ALS patients (sALS, *n* = 28, fALS, *n* = 27), with a mean age of 58.3 ± 11.5 years and 19 HC with a mean age of 52.8 ± 17.5 years (*P* = 0.128). The demographic and clinical features of ALS patients are summarized in Table 1. FALS were carriers of a HRE in *C9orf72* (*n* = 13) and mutations in *SOD1* (*n* = 11) and *TARDBP* (*n* = 3).

**Table 1:** Demographic and clinical features of ALS cohort

	Total (*n* = 55)	sALS (*n* = 28)	fALS (*n* = 27)	*P*-value	*C9orf72* (*n* = 13)	*SOD1* (*n* = 11)	*TARDBP* (*n* = 3)	*P*-value
Age, yeas	58.3 ± 11.5	63.9 ± 11	52.5 ± 9	< 0.0001	54.6 ± 6.2	48.6 ± 10.8	57 ± 7.8	0.166
Female sex, *n* (%)	20 (36.4)	11 (39.3)	9 (33.3)	0.646	4 (30.8)	3 (27.3)	2 (66.6)	0.250
Age at onset, yeas	56.8 ± 11	62 ± 10.5	51.4 ± 8.8	0.0002	53.6 ± 5.9	47.6 ± 11.1	55.3 ± 6.7	0.183
Spinal phenotype, *n* (%)	38 (69.1)	17 (60.7)	21 (77.8)	0.171	8 (61.5)	10 (90.9)	3 (100)	0.139
Disease duration, months	13 [9–24]	13 [8.3–24]	16 [10–25]	0.838	10 [7–14]	25 [12–42]	16 [16–32]	0.004

Demographic and clinical features of enrolled sALS and fALS patients. Mean ± SD or median [IQR] and number (%), as appropriate

*fALS* familial amyotrophic lateral sclerosis, *sALS* sporadic amyotrophic lateral sclerosis

**Fig. 7:**
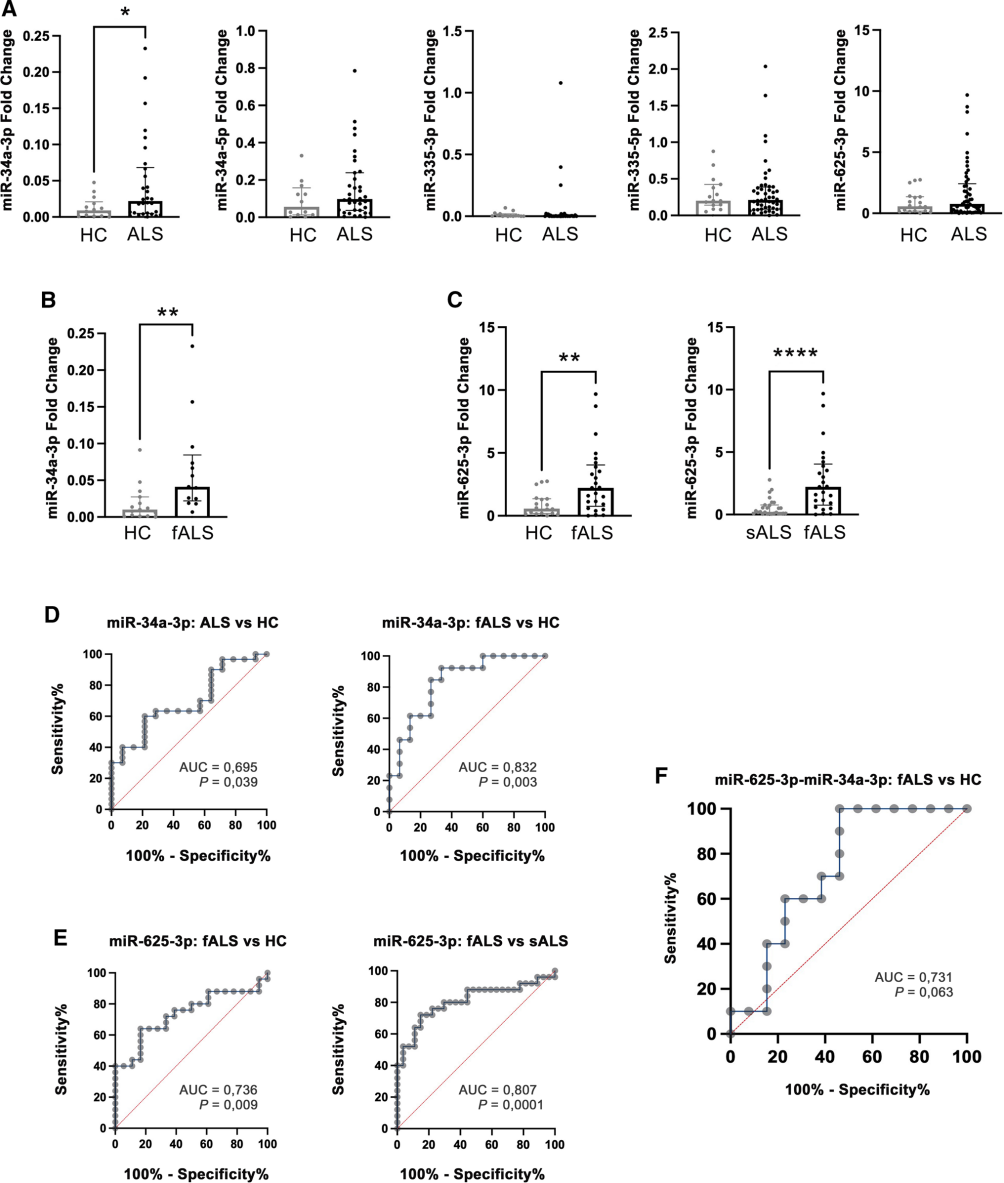
Distribution of miRNA levels in CSF of ALS patients. **A** CSF miR-34a-3p, miR-34a-5p, miR-335-3p, miR-335-5p and miR-625-3p levels in healthy controls (HC) and ALS patients. CSF miR-34a-3p levels are higher in ALS than in HC (*P < 0.05). Scatter dot plot values represent medians and [IQR]. **B** CSF miR-34a-3p levels are increased in familial ALS (fALS) compared to HC (**P < 0.01). **C** CSF miR-625-3p levels are higher in fALS compared to HC (**P < 0.01) and in fALS compared to sporadic ALS (sALS) (****P < 0.001). **D** ROC curve showed accuracy of CSF miR34a-3p levels in ALS vs. HC (*P < 0.05) and fALS vs. HC (**P < 0.01). **E** ROC curve showed accuracy of CSF miR-625-3p levels in fALS vs. HC (**P < 0.01) and in fALS vs. sALS (***P < 0.001). **F** ROC curve showed accuracy of CSF miR-625-3p/miR-34a-3p pair in fALS vs. HC (non-significant)

We compared CSF miRNA levels between HC and ALS patients and we found increased CSF miR-34a-3p levels in ALS with respect to controls (*P* = 0.039), while other miRNA concentrations did not significantly differ between the two groups (Fig. 7A). Then, we performed between-group comparisons and we found that CSF miR-34a-3p levels were higher in fALS compared to HC (*P* = 0.0022) (Fig. 7B). Moreover, miR-625-3p concentrations were increased in fALS patients as compared with HC (*P* = 0.0084) and sALS patients (*P* < 0.0001) (Fig. 7C).

To test the reliability of CSF miRNAs in identifying ALS subgroups, we performed ROC analysis and binomial logistic regression analyses. ROC analysis showed that miR-34a-3p was able to accurate discriminate ALS (AUC 0.695, *P* = 0.039) and fALS (AUC 0.832, *P* = 0.003) from HC (Fig. 7D) and provided a cutoff value of 0.0188 (sensitivity 60%, specificity 78.6%) and of 0.0159 (sensitivity 92.3%, specificity 66.7%), respectively. CSF miR-34a-3p concentrations higher than 0.0188 predicted ALS diagnosis compared to healthy controls in a binomial regression analysis with an OR = 3.667 (95%CI = 1.064–14.15, *P* = 0.046). CSF miR-34a-3p levels higher than 0.0159 were significantly associated with fALS (OR = 20, 95%CI = 2.86–413.9, *P* = 0.009, Fig. 7D). ROC analysis for CSF miR-625-3p levels showed a moderate accuracy in distinguishing fALS from HC (AUC 0.736, *P* = 0.009) and fALS from sALS group (AUC 0.807, *P* = 0.0001) (Fig. 7E), yielding a cutoff value of 1.462 (sensitivity 64%, specificity 83.3%) and 1.051 (sensitivity 72%, specificity 85.2%), respectively. In a binomial logistic regression analysis, CSF miR-625-3p levels higher than cutoff values were associated with fALS with an OR = 4.653 (95%CI = 1.35–17.63, *P* = 0.018) compared to HC and with fALS with an OR = 11.33 (95%CI = 3.237–48.43, *P* = 0.0004) in comparison with sALS group (Fig. 7E). Finally, we performed a ROC analysis between fALS and HC by using the ratio of levels of miR-625-3p/miR-34a-3p pair. This shows an AUC of 0.731 with a *P* = 0.063 in discriminating fALS from HC (Fig. 7F).

## Discussion

In the past years, several strategies have been developed to understand both the genetic and molecular mechanisms of ALS, in the attempt to accelerate the discovery of effective treatments [42–45]. Although the pathological events underlying ALS have not been completely clarified yet, defective RNA metabolism is known to be deeply associated with the pathology [46]. Particularly, aberrant miRNA biogenesis has already been related to stress response induced by mutations in the *TARDBP*, *FUS* and *SOD1* genes, providing a potential link between defective miRNA biogenesis and ALS [29, 47, 48]. Interestingly, several studies have already reported a general dysregulation of miRNA expression in both fALS and sALS cases, suggesting that altered miRNA expression could be a common molecular denominator of multiple forms of ALS [28, 49–55].

We performed miRNA expression profile analysis of iPSC-derived MNs from fALS patients and we demonstrated that miR-34a (3p and 5p) and miR-335 (3p and 5p) were commonly dysregulated in *C9orf72*-, *SOD1*- and *TARDBP*-MNs. Bioinformatic analysis showed that these miRNAs regulate several genes associated with programmed cell death, synaptic plasticity and mitochondrial biogenesis, biological pathways/processes which well correlate with the disease pathogenesis [56]. Indeed, since miRNAs are fundamental for ensuring the physiological homeostasis of tissues, alterations in their expression profile can result in massive impairment of multiple biological pathways [57–59].

We have already described a downregulation of miR-34a expression in iPSC-derived MN progenitors derived from both sALS and fALS patients, supporting its putative role in cell cycle regulation, induction of apoptosis after cell damage and autophagy [53]. Notably, miR-34a is involved in neuronal differentiation and neurogenesis [60] and dysregulation of its expression results in early neurodegeneration in SOD1 mice [61, 62]. An alteration of miR-34a levels has also been reported in monocytes from ALS patients and mouse models, supporting its involvement in neurodegenerative disorder-related inflammation [37]. Recent evidence has shown that in neurodegenerative diseases, a group of miRNAs, including miR-34a and miR-335, enhances reactive oxygen species generation, perturbing the function of mitochondrial antioxidative enzymes [63]. Moreover, data from ALS subjects’ serum revealed a strong reduction in miR-335-5p levels, which correlates with neuronal mitochondrial dysfunction and apoptosis [64]. The role of miR-335-5p in neurodegeneration is further supported by the evidence that downregulation of expression is necessary to maintain hippocampal synaptic plasticity and spatial memory processes in mice [65].

We also assayed the miRNA profiles of exosomes isolated from the culture medium of ALS and healthy MN cultures and we identified a dysregulation of ex-miR-625-3p in ALS-MNs. Indeed, exosomes seem to have a key role in intercellular communication, potentially promoting the progression and the spread of neurodegenerative disorders by modulating cell proliferation, neuronal stability, inflammation and immune response [23, 66]. Bioinformatics analysis revealed that miR-625-3p is predicted to be associated with cell-to-cell communication, autophagy and immune system pathways. Interestingly, miR-625-3p has been identified as a target of long noncoding RNA-p21, which mediates neuroinflammation, oxidative stress, apoptosis and neuronal death [67, 68]. Increased levels of miR-625-3p have been also reported in the muscle tissue of ALS patients [69].

Modulation of one or more miRNAs could be a potential therapeutic strategy. Indeed, restoring the miRNA balance may be particularly interesting since they can modulate multiple pathways simultaneously, but may interact with off-target genes. We successfully used synthetic sequences that mimic endogenous miRNAs to functionally increase the levels of deregulated miRNAs in iPSC-derived MNs. However, our data showed that the modulatory effect of miRNA mimics was not efficacious in rescuing the expression of the target genes, suggesting that changes in target mRNA levels in affected MNs could not be merely explained by differences in miRNA amount. Alternatively, the treatment needs to be extended in time to permanently change gene target expression or through a direct modulation of crucial target genes. Overall, these experiments together with bioinformatic findings could provide useful insights into specific deregulated pathways in ALS that can represent the ground to understand the pathogenesis and find new therapeutic targets.

There has been an increasing interest in investigating miRNAs in biological fluids as potential disease biomarkers in neurodegenerative disorders. Indeed, they showed exceptional stability in body fluids which allows accurate measurement of their expression levels [70]. Notably, the CSF may be the most promising biological fluid for deep investigation of the pathomechanisms underlying neurodegeneration due to its proximity to the CNS [30]. Different studies have already identified specific subsets of dysregulated miRNAs in serum and CSF samples of patients compared to controls [12, 31, 32, 71–73]. Our data showed an upregulation of CSF miR-34a-3p expression in ALS patients compared to healthy subjects, which has been confirmed by ROC analysis. FALS patients showed significantly higher CSF levels of both miR-34a-3p and miR-625-3p, arguing in favor of the role of these miRNAs in disease pathogenesis. Moreover, miR-625-3p was increased in fALS compared to sALS, suggesting that familial forms might share different pathological mechanisms from sporadic cases. In our cohort, we did not find any significant differences in CSF levels of miR-335-3p and miR-335-5p between ALS and controls, although other studies reported that plasma miR-335 levels accurately distinguish patients with neurodegenerative disorders such as FTD and ALS from controls [74]. Finally, ROC and regression analyses allowed to identify cutoff values for CSF miR-34a-3p and miR-625-3p able to discriminate fALS from both healthy subjects and sporadic patients. Combined analysis using the miR-625-3p/miR-34a-3p pair did not reach significance for discrimination of fALS from controls, likely due to the relatively small sample size. Future studies on broader populations might strengthen the power of these findings and explore the potentiality of these miRNAs as disease biomarkers.

## Conclusions

This study shed light on the common pathological mechanisms underlying MN degeneration, confirming the relevance of miRNA modulation in ALS pathogenesis and paving the way for the development of miRNA-based therapeutic approaches aimed at modifying disease pathogenesis. Moreover, we demonstrated that analyzing miRNAs present in CSF could represent a promising tool to define the classification, prognosis, and progression in the context of ALS and eventually in other neurodegenerative diseases.

## Supplementary Information

Below is the link to the electronic supplementary material.

Supplementary file1 Figure S1: iPSC lines generation and differentiation into MNs. (A) Both controls and ALS-iPSCs expressed the pluripotency markers OCT4, SOX2 and SSEA4. (B) iPSC-derived MNs from ALS and healthy subjects showed typical MN markers such as SMI32, TUBB3 and HB9. Nuclei are stained with DAPI, blue. (TIF 7436 KB)

Supplementary file2 Table S1: List of selected raw data from TLDA experiments. Expression levels of miR-34a-3p, miR-34a-5p, miR-335-3p, miR-335-5p and miR-625-3p in ALS-MNs (A) and exosomes isolated from ALS-MNs (B). (DOCX 18 KB)

Supplementary file3 Table S2: Pathway analysis results derived from Reactome analysis on the 21 target genes identified in ALS-MNs. The table shows all identified pathways, sorted by P-value. 16 out of 21 genes were found in Reactome, with 343 pathways hit by at least one of them. FAM208A, FOXN3, KLHDC10, NUFIP2 and UBAP2 genes were neither found nor mapped to any entity in Reactome. (XLSX 64 KB)

Supplementary file4 Table S3: Pathway analysis results derived from Reactome analysis on the 15 target genes of ex-miR-625-3p. The table shows all the identified pathways, sorted by P-value. 10 out of 15 genes were found in Reactome and 148 pathways were hit by at least one of them. FKTN, INIP, PAPD5, PEG10 and VEZT genes were not found or mapped to any entity in Reactome. (XLSX 37 KB)

## Data Availability

All data generated or analysed during this study are included in this published article [and its supplementary information files].

## Abbreviations

ALS: Amyotrophic lateral sclerosis
CNS: Central nervous system
MNs: Motor neurons
sALS: Sporadic amyotrophic lateral sclerosis
fALS: Familial amyotrophic lateral sclerosis
miRNAs: MicroRNAs
ex-miRNAs: Exosomal microRNAs
CSF: Cerebrospinal fluid
EVs: Extracellular vesicles
FTD: Frontotemporal dementia
HRE: Hexanucleotide repeat expansion
ALSFRS-R: Amyotrophic Lateral Sclerosis Functioning Rating Scale revised
GOI: Genes of interest
HC: Healthy controls
ROC: Relative operating curve
AUC: Area under the curve
