# Supplementary figures and images for: Insights into the identification of a molecular signature for amyotrophic lateral sclerosis exploiting integrated microRNA profiling of iPSC-derived motor neurons and exosomes
Source: Cell Mol Life Sci. 2022 Mar 14;79(3):189. doi: 10.1007/s00018-022-04217-1 (PMC8921154; doi:10.1007/s00018-022-04217-1)

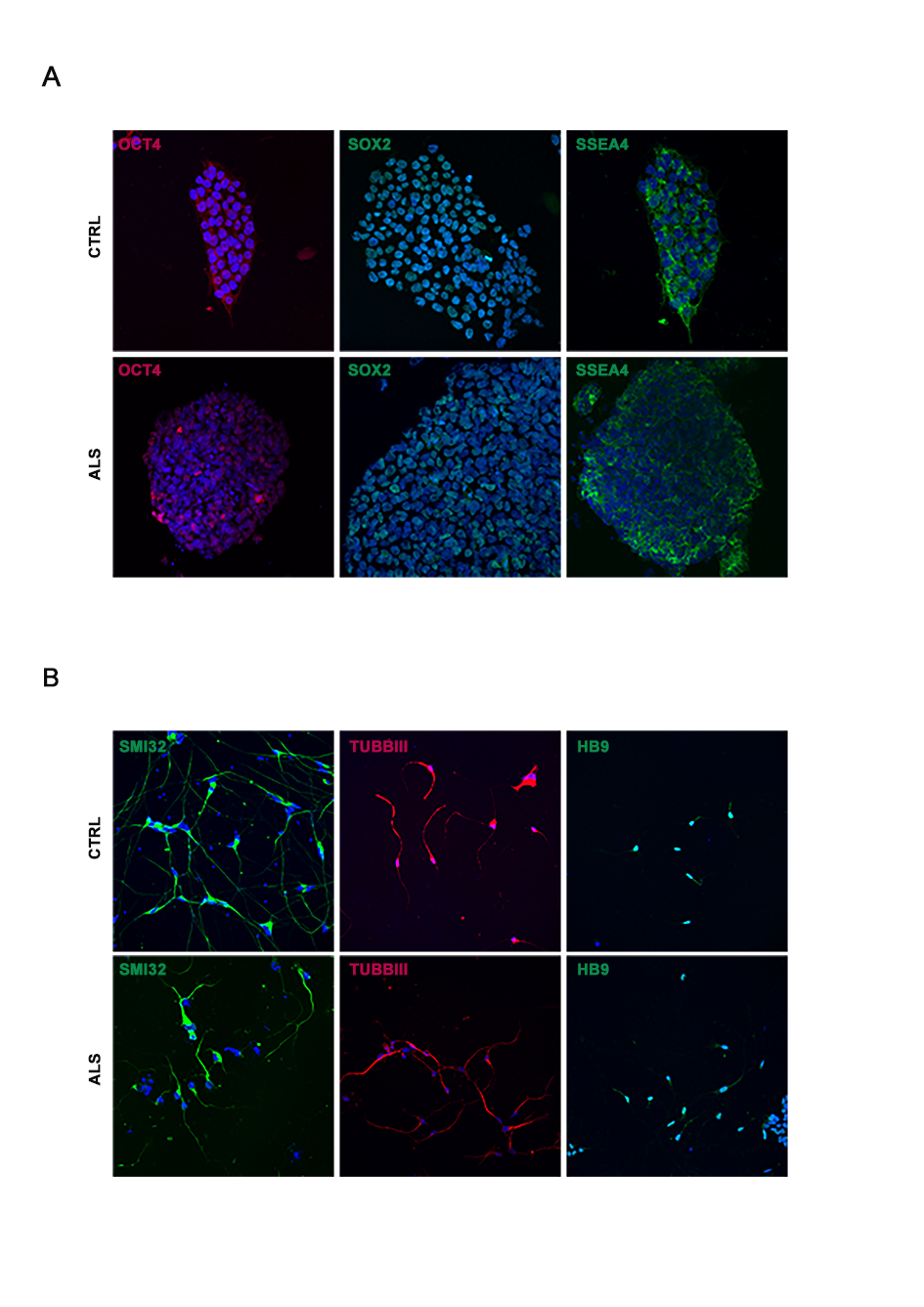

Supplement: Supplementary file 1 — Supplementary file1 Figure S1: iPSC lines generation and differentiation into MNs. (A) Both controls and ALS-iPSCs expressed the pluripotency markers OCT4, SOX2 and SSEA4. (B) iPSC-derived MNs from ALS and healthy subjects showed typical MN markers such as SMI32, TUBB3 and HB9. Nuclei are stained with DAPI, blue. (TIF 7436 KB) [file 18_2022_4217_MOESM1_ESM.tif]
