# Supplementary material for: Insights into the identification of a molecular signature for amyotrophic lateral sclerosis exploiting integrated microRNA profiling of iPSC-derived motor neurons and exosomes
Source: Cell Mol Life Sci. 2022 Mar 14;79(3):189. doi: 10.1007/s00018-022-04217-1 (PMC8921154; doi:10.1007/s00018-022-04217-1)
Supplement: Supplementary file 2 — Supplementary file2 Table S1: List of selected raw data from TLDA experiments. Expression levels of miR-34a-3p, miR-34a-5p, miR-335-3p, miR-335-5p and miR-625-3p in ALS-MNs (A) and exosomes isolated from ALS-MNs (B). (DOCX 18 KB) [file 18_2022_4217_MOESM2_ESM.docx]

**A**

| **Biological group** | **miRNA** | **RQ** | **p-Value** |
| --- | --- | --- | --- |
| *C9orf72*-MNs | hsa-miR-34a-3p | 0,780 | 0,273 |
|  | hsa-miR-34a-5p | 0,420 | 0,013 |
|  | hsa-miR-335-3p | 0,328 | - |
|  | hsa-miR-335-5p | 0,150 | 0,017 |
|  | hsa-miR-625-3p | 0,488 | 0,197 |
| *SOD1*-MNs | hsa-miR-34a-3p | 0,155 | 0,136 |
|  | hsa-miR-34a-5p | 0,130 | 0,010 |
|  | hsa-miR-335-3p | 0,676 | 0,000 |
|  | hsa-miR-335-5p | 0,353 | 0,300 |
|  | hsa-miR-625-3p | 0,436 | 0,539 |
| *TARDBP*-MNs | hsa-miR-34a-3p | 0,238 | 0,041 |
|  | hsa-miR-34a-5p | 0,151 | 0,083 |
|  | hsa-miR-335-3p | 0,043 | 0,036 |
|  | hsa-miR-335-5p | 0,017 | 0,021 |
|  | hsa-miR-625-3p | 1,095 | 0,971 |

**B**

| Biological group | ex-miRNA | RQ | p-Value |
| --- | --- | --- | --- |
| *C9orf72*-exosomes | hsa-miR-34a-3p | 0,635 | 0,390 |
|  | hsa-miR-34a-5p | 0,875 | 0,857 |
|  | hsa-miR-335-3p | 0,311 | - |
|  | hsa-miR-335-5p | 0,151 | 0,429 |
|  | hsa-miR-625-3p | 0,363 | 0,044 |
| *SOD1*-exosomes | hsa-miR-34a-3p | 0,090 | 0,137 |
|  | hsa-miR-34a-5p | 0,188 | 0,070 |
|  | hsa-miR-335-3p | 0,174 | 0,467 |
|  | hsa-miR-335-5p | 0,350 | 0,482 |
|  | hsa-miR-625-3p | 0,069 | 0,050 |
| *TARDBP*-exosomes | hsa-miR-34a-3p | 0,100 | 0,130 |
|  | hsa-miR-34a-5p | 0,226 | 0,209 |
|  | hsa-miR-335-3p | - | - |
|  | hsa-miR-335-5p | 0,049 | - |
|  | hsa-miR-625-3p | 0,176 | 0,258 |
